# Bcl11b is essential for licensing Th2 differentiation during helminth infection and allergic asthma

**DOI:** 10.1038/s41467-018-04111-0

**Published:** 2018-04-26

**Authors:** Kyle J. Lorentsen, Jonathan J. Cho, Xiaoping Luo, Ashley N. Zuniga, Joseph F. Urban, Liang Zhou, Raad Gharaibeh, Christian Jobin, Michael P. Kladde, Dorina Avram

**Affiliations:** 1 Department of Medicine, Division of Pulmonary Medicine, College of Medicine, University of Florida, 1600 SW Archer Rd, Gainesville, FL 32610 USA; 2 Department of Anatomy and Cell Biology, University of Florida College of Medicine, Gainesville, FL 32610 USA; 3 Beltsville Human Nutrition Research Center, Agricultural Research Service, Diet, Genomic and Immunology Laboratory, US Department of Agriculture, Beltsville, MD 20705 USA; 4 Department of Infectious Diseases and Immunology, College of Veterinary Medicine, University of Florida, 2015 SW 16th Ave, Gainesville, FL 32608 USA; 5 UF Health Cancer Center, University of Florida, Gainesville, FL 32610 USA; 6 Department of Medicine, Division of Gastroenterology, College of Medicine, University of Florida, 2033 Mowry Rd., CGRC 461, Gainesville, FL 32610 USA; 7 Department of Biochemistry and Molecular Biology, College of Medicine, University of Florida, 2033 Mowry Rd., CGRC 359, Gainesville, FL 32610 USA

## Abstract

During helminth infection and allergic asthma, naive CD4^+^ T-cells differentiate into cytokine-producing Type-2 helper (Th2) cells that resolve the infection or induce asthma-associated pathology. Mechanisms regulating the Th2 differentiation in vivo remain poorly understood. Here we report that mice lacking Bcl11b in mature T-cells have a diminished capacity to mount Th2 responses during helminth infection and allergic asthma, showing reduced Th2 cytokines and Gata3, and elevated Runx3. We provide evidence that Bcl11b is required to maintain chromatin accessibility at Th2-cytokine promoters and locus-control regions, and binds the *Il4* HS IV silencer, reducing its accessibility. Bcl11b also binds *Gata3*-intronic and downstream-noncoding sites, sustaining the* Gata3* expression. In addition, Bcl11b binds and deactivates upstream enhancers at *Runx3* locus, restricting the Runx3 expression and its availability to act at the *Il4* HS IV silencer. Thus, our results establish novel roles for Bcl11b in the regulatory loop that licenses Th2 program in vivo.

## Abstract (web summary)

Th2 cells are critical in the resolution of helminth infection and allergy. Here the authors show that Bcl11b is required to license the Th2 program of helper T cell differentiation and restrict alternate lineage gene expression using in vivo models of helminth infection and asthma.

## Introduction

The molecular pathways dictating effector cell differentiation from naive CD4^+^ T-cells are controlled by transcription factors that regulate the expression of lineage-specific genes. Several of these transcription factors act as pioneers and initiate large scale changes in genetic programs by altering the chromatin landscape to create accessible regions at promoters, enhancers, and locus-control regions (LCRs)^1^. Type-2 T-helper (Th2) cells are formed following the activation of naive CD4^+^ T-cells in the presence of IL-4, and are critical in helminth infections and allergic diseases including asthma^2^. IL-4 is known to activate the signal transducer and activator of transcription 6 (STAT6)^3^, which in turn induces expression of GATA3, a potent pioneer transcription factor that acts at the Th2-LCR, and Th2-cytokine promoters^4^. By enhancing the expression of IL-4, GATA3 enforces a positive feedback loop that stabilizes the Th2 lineage^2^. However, compared to the other T-helper effector lineages, our understanding of the mechanisms behind Th2 differentiation in vivo is incomplete. The role of the canonical IL-4/STAT6 pathway, which has been used in in vitro CD4^+^ T-cell polarization for many years, generated conflicting reports in vivo^5,^ and STAT6-independent mechanisms of Th2 differentiation have been identified^4^.

The Th2 cytokine locus, which contains the *Il4*, *Il5*, and *Il13* genes, is under the control of an LCR located within the 3′ end of the *Rad50* gene^6^. In vivo-deletion studies have shown that mice lacking the Th2 LCR have significantly impaired Th2 cytokine secretion and do not develop severe asthma^7^. The Th2 LCR contains four functionally distinct DNase hypersensitive sites (HSs), of which three are Th2 specific: *Rad50* (R) HS IV, V, and VII. RHS VII has been shown to be critical in forming a poised-chromatin structure, which initiates the long-range interactions between the LCR and the Th2-cytokine promoters^8^. RHS IV needs to have a transcriptionally active configuration promoted by SATB1^9^, while RHS V is needed to enhance the* Il4* transcription through interactions with the *Il4* promoter mediated by GATA3, OCT-1, and ETS-1^10^. In addition to the LCR, Th2 differentiation is controlled by a conserved silencer, downstream of the *Il4* gene at the HS IV^11^. During Th1 differentiation, the transcription factor Runx3 associates with the *Il4* HS IV silencer to block *Il4* transcription^12,13^. In addition, Runx3 attenuates the activity of GATA3 through direct interaction^14^.

Bcl11b functions both as a transcriptional repressor, when associated with the Nucleosome Remodeling and Deacetylase (Mi-2/NuRD) complex^15–17^, and as a transcriptional activator, when associated with the p300 histone acetyl transferase^18^. Bcl11b is expressed in thymocytes starting at the DN2 stage, playing major roles in the commitment to T-cell lineage. It further controls the beta and positive selection of thymoctes^19–23^ and is critical for the development of T-regulatory cells and iNKT cells^24–26^ (and reviewed in ref. ^27^). Bcl11b also controls cytotoxic T-cell function in bacterial and viral infections^28,29^, and is expressed in naive and effector CD4^+^ T-cells^23,28^. Bcl11b blocks GATA3 and IL4 in pathogenic Th17 cells during experimental autoimmune encephalomyelitis (EAE), thus controlling the plasticity of Th17 cells^30^. Bcl11b is also critical for type-2 innate lymphoid cell (ILC2s) development^31,32^, maintenance of their program and identity, as well as for the repression of type-3 ILC program in ILC2s^33^.

Here, we ascertain a new role for Bcl11b in the network of transcription factors that control differentiation of the Th2 lineage in vivo. We identified major defects in the capacity of Bcl11b-deficient T-helper cells to differentiate into Th2 cells in vivo, causing diminished responses to helminth infection and reduced severity of asthma. By evaluating the genome-wide binding of Bcl11b and comparing the changes in the transcriptome and chromatin accessibility, we established that Bcl11b-deficient T-helper cells fail to upregulate GATA3, express Runx3, and have enhanced chromatin accessibility at the *Il4* HS IV silencer, but reduced accessibility at Th2-cytokine LCR and Th2-cytokine promoters. We position Bcl11b as a direct negative regulator of *Runx3*-gene expression, by binding and blocking two upstream enhancers in Th2 cells and naive CD4^+^ T-cells. In addition, we found the Bcl11b binding to conserved *cis*-regulatory sites located in the introns and downstream to the *Gata3* locus. Thus, the reduction in GATA3, combined with increased Runx3 activity at the accessible *Il4* HS IV silencer and diminished IL-4 expression in the absence of Bcl11b, resulted in diminished chromatin opening at the Th2 LCR, and at the *Il13* and *Il5* promoters, followed by reduced Th2 cytokine expression. This cements Bcl11b as an important transcription factor in Th2 lineage licensing. In addition, Bcl11b-deficient Th2 cells showed abnormal expression of alternate-lineage genes, including those of Th17, Th1, and innate cells.

## Results

### Removal of Bcl11b in T-cells attenuates allergic asthma

Our previous studies have shown that in mice induced with EAE, the absence of Bcl11b in mature T-cells caused increased plasticity of Th17 cells by acquisition of GATA3 and IL-4^30^. However, we found that Bcl11b plays a critical role in ILC2s, promoting their program and preserving their identity^33^. Given these results, we investigated the role of Bcl11b during a house dust mite (HDM)-induced asthma, a Th2-mediated disease. For these studies, we used *Bcl11b*^*fl/fl*^ dLck-iCre mice, whose Cre-recombinase is under the distal *Lck* promoter, which becomes active only in mature T-cells^34^. We have previously shown that this late-deleting-Cre system removes Bcl11b in ~80% of the mature CD4^+^ T-cells, and does not cause the removal of Bcl11b in DP and single-positive thymocytes, allowing T-cells to reach normal maturity^28^.

**Fig. 1:**
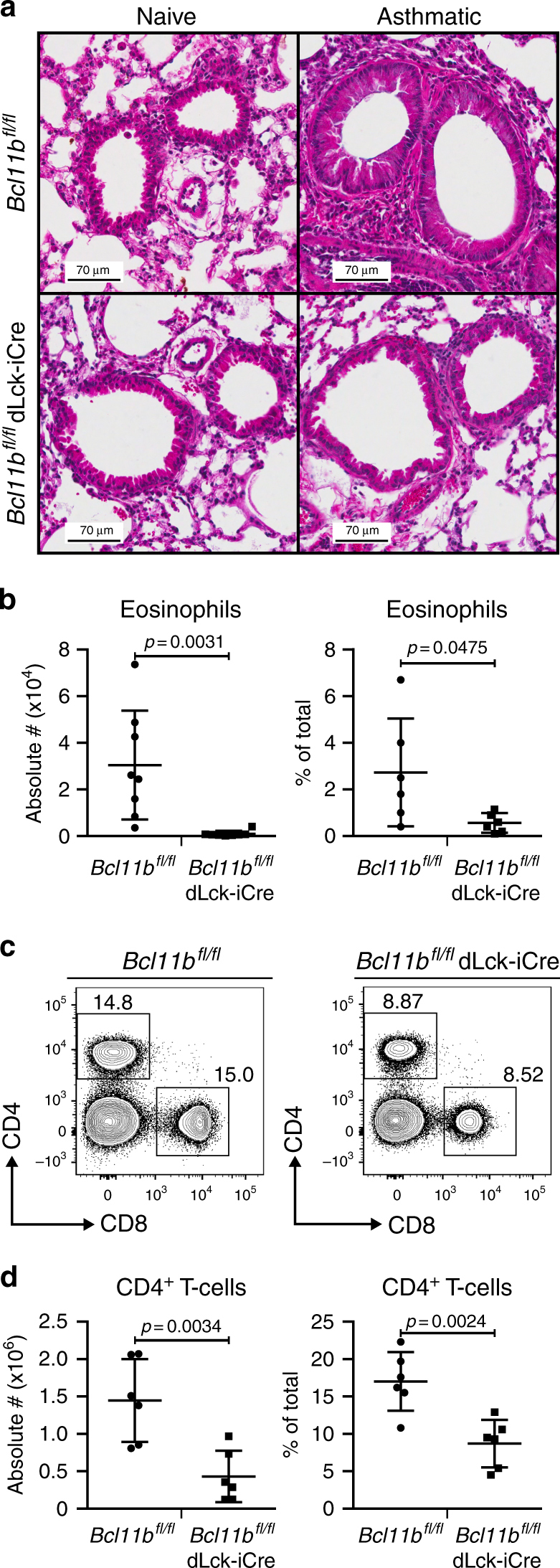
*Bcl11b*^*fl/fl*^ dLck-iCre mice have reduced pulmonary inflammation during allergic asthma. **a** H&E staining of the lung parenchyma sections from asthmatic (*n* = 4) and naive mice (*n* = 2) 29 days after the initial HDM sensitization. Data are representative of two independent experiments. **b** Absolute number (left panel) and frequencies (right panel) of eosinophils in the lung parenchyma of HDM-asthma-induced* Bcl11b*^*fl/fl*^ (*n* = 8) mice and *Bcl11b*^*fl/fl*^ dLck-iCre (*n* = 6) mice. Eosinophils were identified by gating, presented in Supplementary Figure 1b. Data are representative of three independent experiments. **c** Flow cytometry analysis of CD4^+^ and CD8^+^ T-cell populations in the lung parenchyma of the indicated groups of HDM-asthma-induced mice. **d** Absolute number (left panel) and frequencies (right panel) of CD4^+^ T-cells in the lung parenchyma of HDM-asthma-induced *Bcl11b*^*fl/fl*^ (*n* = 8) and *Bcl11b*^*fl/fl*^ dLck-iCre (*n* = 6) mice. Data are representative of three independent experiments. All statistical values in this figure were calculated using Student’s *t*-test. All error bars shown in this figure are of standard deviation

Following sensitization and challenge with HDM extract derived from *Dermatophagoides pteronyssinus* (Supplementary Figure 1a), we found that *Bcl11b*^*fl/fl*^ dLck-iCre mice developed less severe lung pathology than wild-type (WT) mice, with substantially reduced leukocyte infiltration (Fig. 1a). Eosinophils, which are recruited to the lung during HDM-induced asthma, were present in significantly lower numbers in *Bcl11b*^*fl/fl*^ dLck-iCre mice (Fig. 1b and Supplementary Figure 1b). The frequency and absolute numbers of CD4^+^ T-cells were also significantly reduced in the lungs of *Bcl11b*^*fl/fl*^ dLck-iCre mice induced with HDM-asthma (Fig. 1c, d). The frequency of CD4^+^ T-cells within the mediastinal lymph nodes (mediLNs) was also reduced in *Bcl11b*^*fl/fl*^ dLck-iCre mice, however not as severely as in the lung (Supplementary Figure 1c). Taken together, these results demonstrate that the removal of Bcl11b from mature T-cells results in reduced pulmonary pathology after induction of allergic asthma, which was associated with reduced lung eosinophils and CD4^+^ T-cells.

### Absence of Bcl11b in T-cells reduces anti-helminth response

**Fig. 2:**
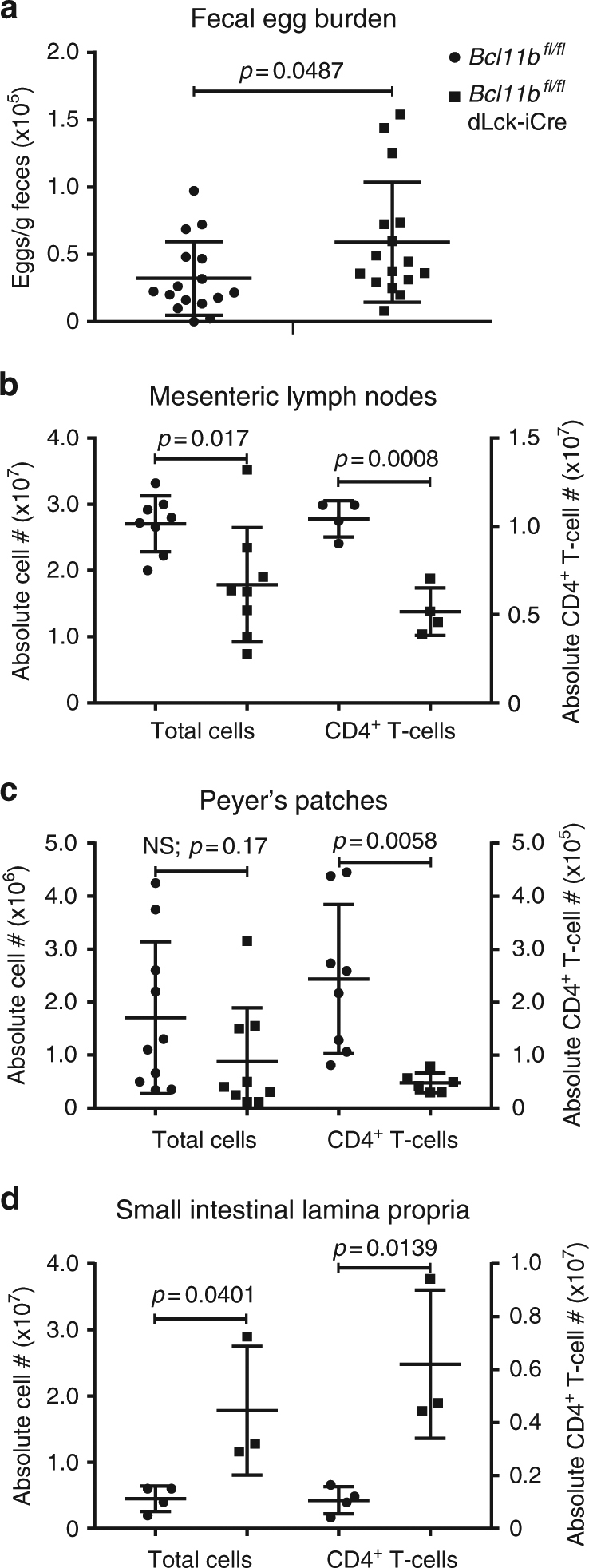
*Bcl11b*^*fl/fl*^ dLck-iCre mice have elevated helminth egg burden in feces following infection with *Heligmosomoides polygyrus bakeri.* **a** Total egg burden in feces of infected *Bcl11b*^*fl/fl*^ dLck-iCre mice (*n* = 16) and *Bcl11b*^*fl/fl*^ control mice (*n* = 16). Data are representative of nine independent experiments. **b** Absolute numbers of total leukocytes (*n* = 8) and CD4^+^ T-cells (*n* = 4) from the mesenteric (m)LNs of helminth-infected mice. **c** Absolute numbers of total leukocytes (*Bcl11b*^*fl/fl*^: *n* = 10; *Bcl11b*^*fl/fl*^ dLck-iCre: *n* = 9) and CD4^+^ T-cells (*Bcl11b*^*fl/fl*^: *n* = 8; *Bcl11b*^*fl/fl*^ dLck-iCre: *n* = 6) from the Peyer’s patches (PPs) of helminth-infected mice from the indicated groups. **d** Absolute numbers of total leukocytes and CD4^+^ T-cells from the small intestine lamina propria (SILP) of helminth-infected *Bcl11b*^*fl/fl*^ (*n* = 4) and *Bcl11b*^*fl/fl*^ dLck-iCre (*n* = 3) mice. Data are representative of three independent experiments. All statistical values in this figure were calculated using Student’s *t*-test. All error bars shown in this figure are of standard deviation

Given the chronic nature of allergic asthma, we investigated the immune response to infection with intestinal helminth *H. polygyrus bakeri*, known to induce a strong primary Th2 response. Unlike many parasitic nematodes that migrate to other mucosal sites, the entire life cycle of *H. polygyrus bakeri* occurs within the gastrointestinal tract^35^. We found that on day 10 post inoculation (p.i.) (Supplementary Figure 2a), *the Bcl11b*^*fl/fl*^ dLck-iCre mice had a significant increase in fecal egg burden, compared to WT mice (Fig. 2a). The Flow cytometry analysis showed a reduction in the absolute numbers of CD4^+^ T-cells in the mesenteric lymph nodes (mLNs) and Peyer’s patches (PPs) of *Bcl11b*^*fl/fl*^ dLck-iCre mice (Fig. 2b, c), however the numbers were increased in the small intestine lamina propria (SILP), as were their relative frequencies (Fig. 2d and Supplementary Figure 2f-g). This increase in SILP CD4^+^ T-cells was found to be related to the upregulation of CCR9 on Bcl11b-deficient CD4^+^ T-cells (Supplementary Figure 2h). Thus, despite the increased abundance of CD4^+^ T-cells in the SILP, the fecal egg burden was higher in *Bcl11b*^*fl/fl*^ dLck-iCre mice compared to WT mice, indicating a less robust Th2 response.

### Bcl11b-deficient T-helper cells downregulate Th2 mRNAs

**Fig. 3:**
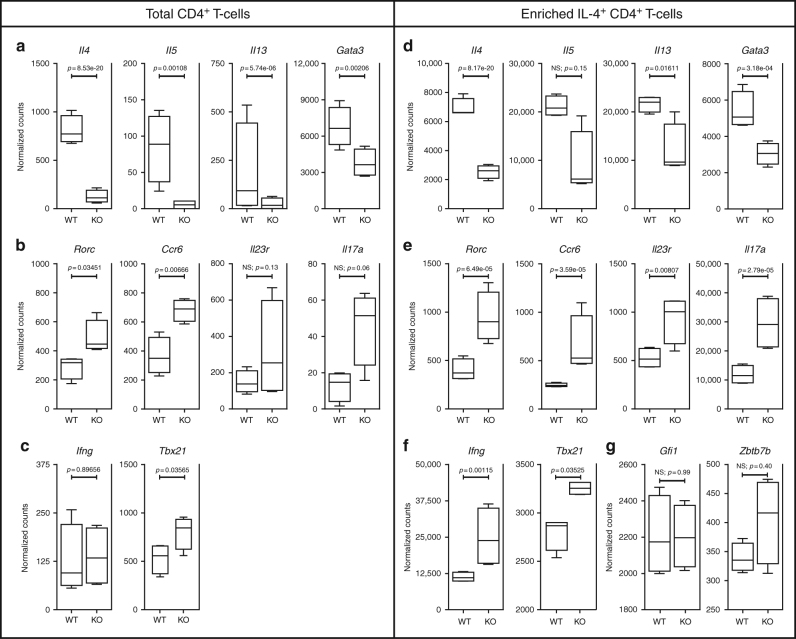
Bcl11b-deficient CD4^+^ T-cells downregulate the Th2 program and upregulate Th17 and Th1 genes. **a**–**c** Box and whisker plots of normalized counts for specific Th2-associated (**a**), Th17-associated (**b**), and Th1-associated (**c**) mRNAs derived from RNA-Seq on total CD4^+^ T-cells from the mLNs of *H. polygyrus bakeri* infected *Bcl11b*^*fl/fl*^ dLck-iCre mice (*n* = 4) and *Bcl11b*^*fl/fl*^ control mice (*n* = 4). Normalized counts and statistical analyses were taken from DESeq2 analysis software. Data are derived from two independent experiments. *p* values have been adjusted for the FDR (false discovery rate) by the Benjamini–Hochberg adjustment. **d**–**f** Box and whisker plots of normalized counts for specific Th2-associated (**d**), Th17-associated (**e**), and Th1-associated (**f**) mRNAs derived from RNA-Seq on IL-4^+^-enriched CD4^+^ T-cells from the lung parenchyma of asthma-induced *Bcl11b*^*fl/fl*^ dLck-iCre mice (*n* = 4) and *Bcl11b*^*fl/fl*^ control mice (*n* = 4). **g** Box and whisker plots of normalized counts for *Gfi1* and *Zbtb7b* mRNA from RNA-Seq on IL-4^+^-enriched CD4^+^ T-cells from the lung parenchyma of asthma-induced *Bcl11b*^*fl/fl*^ dLck-iCre mice (*n* = 4) and *Bcl11b*^*fl/fl*^ control mice (*n* = 4). Normalized counts and statistical analyses were taken from DESeq2 analysis software. *p* values have been adjusted for the FDR (false discovery rate) by the Benjamini–Hochberg adjustment. All error bars shown are of standard deviation

To establish the mechanisms associated with reduced Th2 response in asthmatic and helminth-infected *Bcl11b*^*fl/fl*^ dLck-iCre mice, we next performed RNA-sequencing on CD4^+^ T-cells isolated from: (1) mediLNs of asthma-induced *Bcl11b*^*fl/fl*^ dLck-iCre and WT mice, and (2) mLNs of helminth-infected mice, and found that a large number of mRNAs significantly changed (Supplementary Figure 3). In regard to the Th2 program, we found that the levels of mRNAs for *Gata3* and the Th2 cytokines *Il4, Il5*, and *Il13* were all significantly reduced in the CD4^+^ T-cells from *Bcl11b*^*fl/fl*^ dLck-iCre mice (Fig. 3a). We also detected an increase in several Th17 mRNAs, namely for *Rorc* and *Ccr6*, with *Il17a*, and *Il23r* showing a similar trend (Fig. 3b). Given that *Bcl11b*^*fl/fl*^ dLck-iCre mice show a less robust Th2 response, we further enriched the IL-4-secreting CD4^+^ T-cell population and even in these conditions, the levels of *Il4*, *Il13*, and *Gata3* mRNAs were still significantly reduced in the absence of Bcl11b, with a similar trend in *Il5* mRNA (Fig. 3d). In addition, the previously observed increases in Th17 mRNAs, including for *Rorc*, *Ccr6*, *Il17a*, and *Il23r*, were amplified in the Bcl11b-deficient IL-4^+^ CD4^+^ T-cell population (Fig. 3e). While the levels of *Ifng* transcripts in the total CD4^+^ T-cell population were approximately equal, there was an increase in *Ifng* mRNA levels in the IL-4-enriched CD4^+^ T-cell population of the *Bcl11b*^*fl/fl*^ dLck-iCre mice, compared to WT mice (Fig. 3c, f). *Tbx21* mRNA encoding the Th1 signature transcription factor T-bet was upregulated as well, both in the IL-4-enriched CD4^+^ T-cell population and total CD4^+^ T-cells (Fig. 3c, f). Finally, we found no change in *Gfi1* mRNA (Fig. 3g), known to be downregulated in Bcl11b-deficient ILC2s^33^, or *Zbtb7b* (Fig. 3g), previously found upregulated in Bcl11b-deficient DP thymocytes^36^.

These results, taken together, suggest that, in the context of Th2-mediated diseases, Bcl11b is necessary for Th2 cytokine and *Gata3* gene expression, as well as for the repression of critical Th17 and Th1 cell genes.

### Reduction in Th2 program is caused by intrinsic defects

**Fig. 4:**
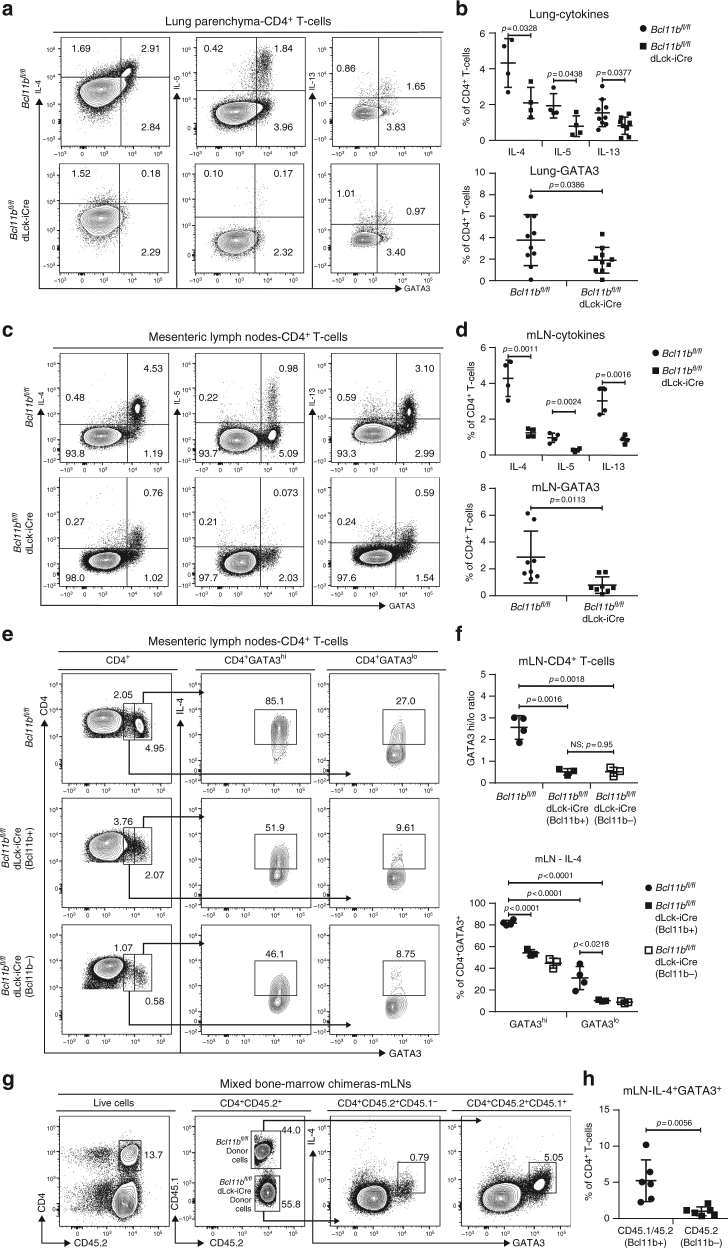
Frequencies of CD4^+^ T-cells expressing Gata3 and Th2 cytokines in asthma and helminth infection are reduced in *Bcl11b*^*fl/fl*^ dLck-iCre mice by an intrinsic defect. **a** Flow cytometry analysis of IL-4 (left column), IL-5 (middle column), and IL-13 (right column) against GATA3 in the CD4^+^ T-cell population of the lung parenchyma of HDM asthma-induced mice. **b** Frequencies of IL-4^+^ (*n* = 4), IL-5^+^ (*n* = 4), and IL-13^+^ (*n* = 8) (top panel) and GATA3^+^ (*n* = 10) (bottom panel) CD4^+^ T-cells from the lungs of the indicated groups. Data are derived from two (IL-4, IL-5, and IL-13 data) or five (GATA3 data) independent experiments. **c** Flow cytometry analysis of IL-4 (left column), IL-5 (middle column), and IL-13 (right column) against GATA3 in the CD4^+^ T-cell population from the mesenteric  (m)LNs of *H. polygyrus* infected mice. **d** Frequencies of IL-4^+^ (*n* = 4), IL-5^+^ (*n* = 4), and IL-13^+^ (*n* = 4) (top panel) and GATA3^+^ (*n* = 8) (bottom panel) CD4^+^ T-cells from mLNs of the indicated groups. Data are derived from two (IL-4, IL-5, and IL-13) or four (GATA3) independent experiments. **e** Flow cytometry analysis of GATA3 in CD4^+^ T-cells (left column) and IL-4 (middle and right columns) in the GATA3^hi^ (middle column) and GATA3^lo^ (right column) CD4^+^ T-cells from the mLNs of *H. polygyrus bakeri* infected mice. **f** The ratio of GATA3^hi^/GATA3^lo^ CD4^+^ T-cells (top) and frequencies of IL-4 secreting cells within the GATA3^hi^ and GATA3^lo^ CD4^+^ T-cells (bottom) from the mLNs of *H. polygyrus bakeri* infected WT (*n* = 4) and *Bcl11b*^*fl/fl*^ dLck-iCre (*n* = 3) mice. Data shown are from two independent experiments. **g** Flow cytometry analysis of IL-4 and GATA3 in CD4^+^ T-cells from the mLNs of mixed bone-marrow chimeric mice infected with *H. polygyrus bakeri*. Cells from WT donors are CD45.1/CD45.2, while cells from *Bcl11b*^*fl/fl*^ dLck-iCre donors are CD45.2. **h** Frequencies of IL-4^+^GATA3^+^ cells from the mLNs of mixed bone-marrow chimeric mice (*n* = 6) infected with *H. polygyrus bakeri*. Data shown are from two independent experiments. All statistical values in this figure were calculated using Student’s *t*-test. All error bars shown in this figure are of standard deviation

We next evaluated the protein levels of GATA3 and Th2 cytokines in the T-helper cells of mice induced with HDM-asthma or infected with helminths. We found that the frequencies of GATA3^+^IL-4^+^, GATA3^+^IL-5^+^, and GATA3^+^IL-13^+^ CD4^+^ T-cells in the lungs of asthma-induced *Bcl11b*^*fl/fl*^ dLck-iCre mice were reduced, as were the frequencies of GATA3^+^, IL-4^+^, IL5^+^, and IL-13^+^ CD4^+^ T-cells. (Fig. 4a, b). When evaluating *H. polygyrus bakeri*-infected mice, we noticed a similar reduction in Th2 cytokines and GATA3 within the CD4^+^ T-cells from the mLNs (Fig. 4c, d) and SILP (Supplementary Figure 4a-c). To further determine the ability of Bcl11b-deficient T-helper cells to differentiate in vivo, we separated the GATA3^+^ CD4^+^ T-cells from the mLNs of helminth-infected mice into GATA3^hi^ and GATA3^lo^ populations, and also gated separately on Bcl11b^−^ and Bcl11b^+^ CD4^+^ T-cells of *Bcl11b*^*fl/fl*^ dLck-iCre mice (Fig. 4e, f). The GATA3^hi^/GATA3^lo^ ratio was 2.6 within the CD4^+^ T-cell population of WT mice (Fig. 4e, f), while this ratio was only 0.52 in the Bcl11b-deficient CD4^+^ T-cell population of *Bcl11b*^*fl/fl*^ dLck-iCre mice (Fig. 4e, f). Even the small population of Bcl11b^+^ “escaper” CD4^+^ T-cells from *Bcl11b*^*fl/fl*^ dLck-iCre mice had a similarly reduced ratio of GATA3^hi^/GATA3^lo^ cells (Fig. 4e, f), likely due to the overall reduction in IL-4. Within the GATA3^hi^ population of WT mice, the frequency of IL4^+^ cells was around 80%. This frequency was reduced by nearly half in both the Bcl11b^−^ and Bcl11b^+^ GATA3^hi^ cells of *Bcl11b*^*fl/fl*^ dLck-iCre mice (Fig. 4e, f). The frequency of IL-4^+^ cells within the GATA3^lo^ cells was only one-third of the GATA3^hi^ cells in the WT mice, and was further reduced in both the Bcl11b^−^ and Bcl11b^+^ cells of *Bcl11b*^*fl/fl*^ dLck-iCre mice (Fig. 4e, f). These results suggest that (1) Bcl11b-deficient CD4^+^ T-cells are unable to completely differentiate into the Th2 lineage, and (2) even those cells that reach high levels of GATA3 cannot produce normal levels of IL-4 in the absence of Bcl11b.

Importantly, the upregulation of the *Rorc* and *Tbx21* mRNAs was not reflected at protein level (Supplementary Figure 5a-d), making it unlikely to contribute to the phenotype. In addition, proliferation of Bcl11b-deficient T-helper cells was not affected, as demonstrated by the similar Ki-67^+^ population within CD4^+^ T-cells from *Bcl11b*^*fl/fl*^ dLck-iCre and WT mice (Supplementary Figure 5c–d).

To establish whether the reduction in Gata3 and IL-4 levels in Bcl11b^−^ CD4^+^ T-cells is cell intrinsic, we generated mixed bone-marrow chimeras. Lethally irradiated recipient CD45.1 mice were reconstituted with cells from *Bcl11b*^*fl/fl*^ (CD45.1/2) and *Bcl11b*^*fl/fl*^ dLck-iCre (CD45.2) mice at a 1:1 ratio. Following reconstitution, the recipient mice were infected with *H. polygyrus bakeri*. Our results show that even in conditions in which Bcl11b^+^ cells are present in equal numbers to Bcl11b^−^ cells, GATA3 and IL-4 levels remained reduced in the Bcl11b^−^ cells (Fig. 4g, h), suggesting that these alterations are intrinsic.

These results demonstrate that in the absence of Bcl11b, CD4^+^ T-cells have an altered ability to differentiate into Th2 lineage properly in vivo, with diminished GATA3 and Th2 cytokine levels, caused by intrinsic defects.

### Bcl11b binding at Th2 cytokine locus and *Gata3*

**Fig. 5:**
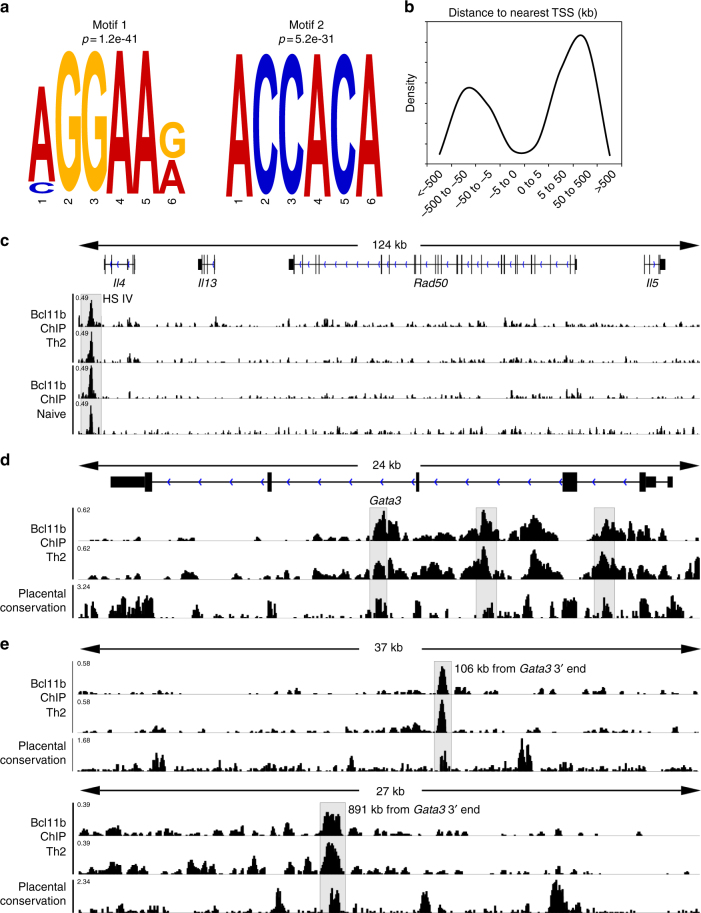
Bcl11b binds at the *Gata3* locus and downstream and at the *Il4* HS IV silencer but not at the Th2-LCR or Th2-cytokine promoters in CD4^+^ T-cells. **a** The top two Bcl11b binding motifs discovered using MEME-ChIP motif analysis software from Bcl11b ChIP-seq of CD4^+^ T-cells from WT mice infected (*n* = 2) with *H. polygyrus bakeri*. **b** Density distribution of Bcl11b binding relative to the nearest transcription start site (TSS). Data obtained from Genomic Regions Enrichment of Annotations Tool (GREAT). **c**–**e** Integrative Genomics Viewer visualizations of Bcl11b ChIP-Seq tracks at the Th2 cytokine locus (c, *Il4* HS IV silencer is highlighted), *Gata3* (**d** intronic regions are highlighted), and (**e** conserved non-coding binding sites downstream of *Gata3* are highlighted) from naive (*n* = 2) (**c**) or helminth-infected (*n* = 2) (**c**–**e**) mice. ChIP-Seq track scale bar normalized to sequences per million reads (SPMR)

To further determine if Bcl11b directly mediates the observed changes in the Th2 program, we performed Bcl11b ChIP-seq on CD4^+^ T-cells from the mLNs of *H. polygyrus bakeri* infected mice. Using the MEME-ChIP motif analysis software^37^, we established the conserved binding motifs for Bcl11b, of which the top two are shown (Fig. 5a). The first identified motif is a known Ets-1 binding motif, while the second motif, identical to a previously published Bcl11b binding site in striatal neuronal cells^38^, is a Runx binding motif^39^. Bcl11b binding sites were found to have increased presence at regions greater than 5 kb from the TSS (Fig. 5b), suggesting that, overall, Bcl11b may normally act through distal regulatory elements, such as enhancers and silencers, rather than through promoters.

Among the Th2 genes, the most striking finding was the fact that Bcl11b did not bind to *Il4, Il13,* or *Il5* promoters or to the Th2 LCR located within *Rad50* gene (Fig. 5c). However, Bcl11b bound to the *Il4* HS IV silencer, located downstream of the 3′ end of the *Il4* gene (Fig. 5c), suggesting that Bcl11b may be necessary to limit the accessibility of *Il4* HS IV silencer in Th2 cells. In addition, we found Bcl11b binding to conserved intronic sites within the *Gata3* locus (Fig. 5c), as well as to other conserved non-coding regions downstream of the *Gata3* locus (Fig. 5e), which suggests that Bcl11b is potentially involved in direct regulation of *Gata3* gene expression.

### Bcl11b regulates chromatin opening at Th2 cytokine locus

**Fig. 6:**
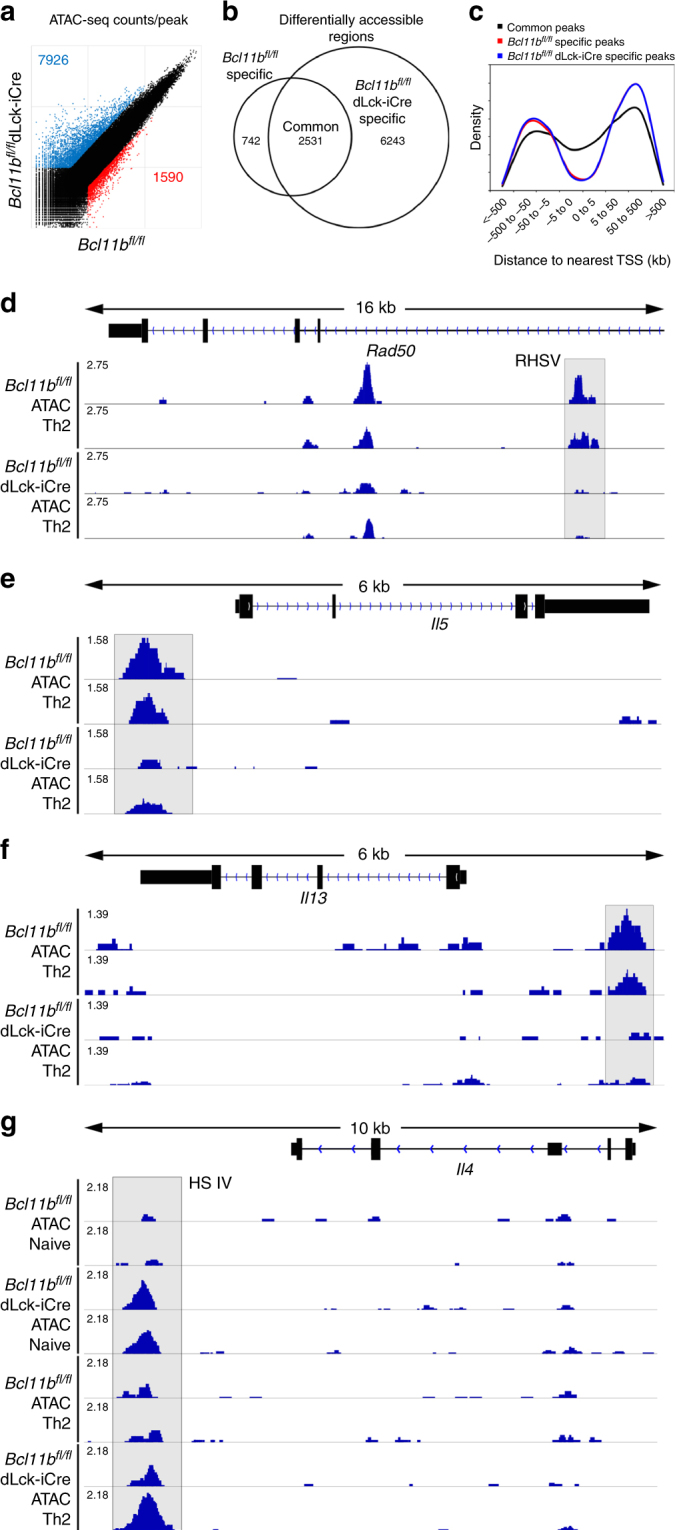
Removal of Bcl11b results in reduced chromatin accessibility at key Th2 loci and increased accessibility at the *Il4* HS IV silencer. **a** Scatterplot of normalized ATAC-Seq coverage per peak. **b** Venn diagram of the distribution of significantly differentially accessible regions. Significance was calculated by MAnorm analysis and was defined as |log2(fold-change)| > 1 and *p* < 0.05. *p* value was adjusted for the FDR (false discovery rate) by the Benjamini–Hochberg adjustment. **c** Density distribution of accessible regions relative to the nearest transcription start site (TSS). Data obtained from Genomic Regions Enrichment of Annotations Tool (GREAT). **d**–**g** Integrative Genomics Viewer visualizations of ATAC-Seq tracks at *Rad50* Th2 LCR (RHS V highlighted) (**d**), *Il5* (promoter highlighted) (**e**), *Il13* (promoter highlighted) (**f**), and *Il4* (*Il4* HS IV silencer highlighted) (**g**). ATAC-Seq track scale bar normalized to SPMR

We next performed the Assay for Transposase-Accessible Chromatin and sequencing (ATAC-seq) to evaluate the changes in chromatin accessibility^40^ in CD4^+^ T-cells isolated from the lung of *Bcl11b*^*fl/fl*^ dLck-iCre and WT mice with HDM-induced asthma (Fig. 6). We identified over 60,000 accessible regions in CD4^+^ T-cells from either WT or *Bcl11b*^*fl/fl*^ dLck-iCre mice. Of these, 9516 were differentially accessible between the two groups (Fig. 6a). The majority of differentially accessible regions (~65%) were unique to Bcl11b-deficient cells, while relatively few (~8%) were unique to WT cells (Fig. 6b). This profile suggests that in these cells, Bcl11b plays a larger role in restricting chromatin accessibility than in opening it. Furthermore, using Genomic Regions Enrichment of Annotations Tool (GREAT)^41^, we found that the differentially accessible regions unique to one group were more likely to be found further away from the TSSs, while those regions common to both groups had a higher probability of being found closer to the TSSs (Fig. 6c). This suggests that Bcl11b is more likely to regulate enhancers and silencers rather than promoters, similarly to what we found for its binding relative to the TSSs (Fig. 5b).

We focused on genes related to Th2 response, and found that the *Rad50* HS 5 (RHS V) within the Th2 cytokine LCR had significantly reduced accessibility in Bcl11b-deficient T-helper cells compared to WT cells from asthma-induced mice (Fig. 6d), despite the fact that Bcl11b did not bind to this region (Fig. 5c). In addition, the accessibility at *Il5* and *Il13* promoters was reduced in the absence of Bcl11b (Fig. 6e, f), again without Bcl11b binding at these promoters (Fig. 5c). However, there was a significant increase in the chromatin accessibility at the *Il4* HS IV silencer both in Th2 cells and in naive CD4^+^ T-cells (Fig. 6g), where Bcl11b was found to bind (Fig. 5c).

These results show that the absence of Bcl11b in T-helper cells in Th2 conditions in vivo causes a failure in chromatin opening at the Th2 LCR, *Il5* and *Il13* promoters, and an inability to reduce the chromatin accessibility at the *Il4* HS IV silencer, including in naive cells.

### Other genes regulated by Bcl11b in Th2 cells

As shown above mRNAs for Th17 and Th1 genes were upregulated in the absence of Bcl11b. While there was no binding of Bcl11b at *Rorc*, *Tbx21, IL23R, and CCR6* loci, Bcl11b did bind to conserved non-coding regions adjacent to *Ifng* and *Il17* loci (Supplementary Figure 6a-b), suggesting a direct mechanism of repression of their expression. In addition, absence of Bcl11b caused an increase in mRNAs for several myeloid genes, including *Itgam* (Cd11b), *Itgax* (Cd11c), *Fcer1g* and *Fcgr3* (Supplementary Figure 6c), and of mRNAs encoding killer cell lectin-like receptors, including *Klrc1* and *Klrd1* (Supplementary Figure 6d). Bcl11b directly associated with non-coding regions in close proximity to these genes (Supplementary Figure 6e), as well as within non-coding regions in the *Spi1* gene, encoding PU.1 transcription factor, which, was however not upregulated at the mRNA level (Supplementary Figure 6f). These results suggest that Bcl11b directly restricts expression of *Ifng* and *Il17* and of several myeloid and NK genes in Th2 cells.

### Bcl11b-deficient T-helper cells have elevated Runx3

**Fig. 7:**
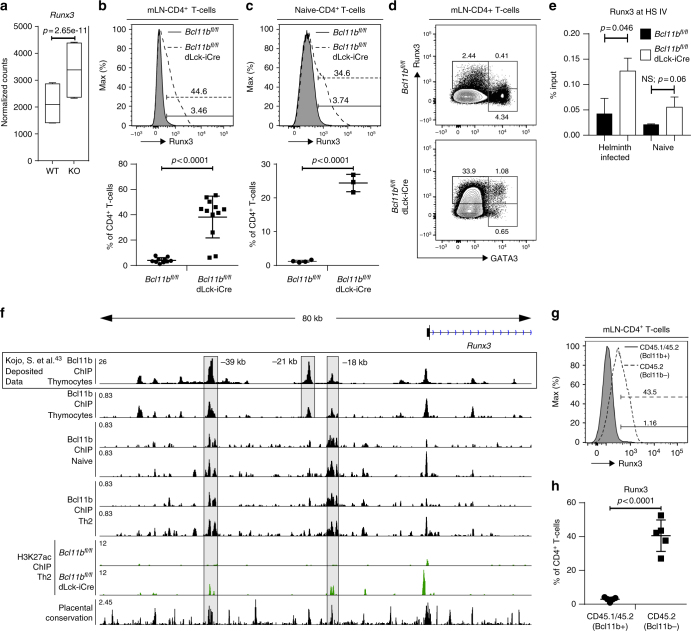
Bcl11b binds to enhancers upstream of *Runx3* locus to restrain *Runx3* expression in CD4^+^ T-cells. **a** Box and whisker plots of normalized counts for *Runx3* from RNA-Seq on CD4^+^ T-cells from the mesenteric (m)LNs of helminth-infected mice. Data (*n* = 4) are derived from two independent experiments. *p* values have been adjusted for the FDR (false discovery rate) by the Benjamini–Hochberg adjustment. **b** Flow cytometry analysis of Runx3 in CD4^+^ T-cells from the mLNs of *H. polygyrus bakeri* infected *Bcl11b*^*fl/fl*^ (*n* = 10) and *Bcl11b*^*fl/fl*^ dLck-iCre (*n* = 12) mice (top) and their relative frequencies (bottom). Data shown are from five independent experiments. **c** Flow cytometry analysis of Runx3 in CD4^+^ T-cells from the mLNs of naive *Bcl11b*^*fl/fl*^ (*n* = 4) and *Bcl11b*^*fl/fl*^ dLck-iCre (*n* = 3) mice (top) and their relative frequencies (bottom). Data shown are from two independent experiments. **d** Flow cytometry analysis of Runx3 vs. Gata3 in total CD4^+^ T-cells from the mLNs of helminth-infected mice. Data representative of three independent experiments. **e** Runx3 enrichment at the HS IV silencer determined by Runx3 ChIP-qPCR performed on CD4^+^ T-cells isolated from the mLNs of helminth-infected mice or the spleen and peripheral lymph nodes of naive mice. ChIP-PCR enrichment is expressed as a percentage of input (*n* = 2). **f** Integrative Genomics Viewer visualizations of Bcl11b ChIP-Seq in total thymocytes (deposited data from Kojo et al.^43^) (top track), Bcl11b ChIP-Seq in CD8-enriched thymocytes from naive mice (second track), Bcl11b ChIP-Seq in naive in CD4^+^ T-cells (tracks 3–4), Bcl11b ChIP-Seq in CD4^+^ T-cells from *H. polygyrus* infected mice (tracks 5–6) and H3K27ac ChIP-Seq (tracks 7–8) in WT and Bcl11b-deficient CD4^+^ T-cells from *H. polygyrus* infected mice at *Runx3* locus. **g** Histogram of Runx3 in CD4^+^ T-cells from the mLNs of mixed bone-marrow chimeric mice infected with *H. polygyrus bakeri*. Gating strategy (shown in Fig. 4g) was first on CD4^+^ T-cells, and then on CD45.1+CD45.2+ (WT) (filled histogram) and CD45.2+ (Bcl11b−) (dashed, open histogram). **h** Frequencies of Runx3^+^ cells in the CD4^+^ T-cells from the mLNs of mixed bone-marrow chimeric mice (*n* = 5) infected with *H. polygyrus bakeri*. Data shown are from two independent experiments. All statistical values in this figure were calculated using Student’s *t*-test except for panel **a**, which was calculated using DESeq2 analysis software. All error bars shown in this figure are of standard deviation

As shown above, absence of Bcl11b caused increased chromatin accessibility at the *Il4* HS IV silencer. In Th1 cells, Runx3 and T-bet synergistically bind to *Il4* HS IV silencer^12^ to repress *Il4* transcription, however Runx3 alone is sufficient to repress *Il4* in Th1 cells^11,12^, even though both Runx3 and T-bet are needed to promote *Ifng* expression. Both *Runx3* and *Tbx21* mRNAs were significantly increased in the absence of Bcl11b (Figs. 3c, f and 7a). However, while T-bet protein remained unchanged (Supplementary Figure 5a), Runx3 protein was considerably upregulated in CD4^+^ T-cells from both helminth-infected and naive *Bcl11b*^*fl/fl*^ dLck-iCre mice (Fig. 7b–d). However Runx3 expression was not upregulated in CD4 SP thymocytes of *Bcl11b*^*fl/fl*^ dLck-iCre mice, and was expressed at equivalent levels to WT mice in CD8 SP thymocytes (Supplementary Figure 7), demonstrating that the phenotypes observed in the periphery are unlikely to be developmental in origin.

Given that Runx3 is known to associate with the *Il4* HS IV silencer to block *Il4* transcription in Th1 cells^12^, we investigated whether the increased Runx3 in the absence of Bcl11b resulted in enhanced Runx3 binding to the *Il4* HS IV silencer by performing Runx3 ChIP-qPCR. We indeed found elevated Runx3 binding to the more accessible *Il4* HS IV silencer in CD4^+^ T-cells from naive and helminth-infected *Bcl11b*^*fl/fl*^ dLck-iCre mice compared to WT (Fig. 7e). Together, these results show that in the absence of Bcl11b, Runx3 levels are elevated, and the available Runx3 binds to the more accessible *Il4* HS IV silencer both in CD4^+^ T-cells of helminth-infected and naive mice.

### Bcl11b directly binds to and represses *Runx3* gene expression

Given the elevated *Runx3* levels, we next examined the Bcl11b ChIP-seq data and found enrichment of Bcl11b binding to two conserved non-coding regions, namely −39 kb and −18 kb upstream of the *Runx3* TSS in CD4^+^ T-cells from naive and helminth-infected mice (Fig. 7f). To further evaluate the chromatin activity at these sites, we performed ChIP-seq for H3K27ac, a histone modification known to be associated with active enhancers and promoters of actively transcribed genes^42^. Absence of Bcl11b caused increased H3K27ac at both the −39 kb and −18 kb regions (Fig. 7f), suggesting that Bcl11b directly restricts expression of *Runx3* by binding to and deactivating upstream *Runx3* conserved enhancers in CD4^+^ T-cells. Further supporting our results is a recent publication in which deletion of the −39 kb *cis*-regulatory region bound by Bcl11b diminished the activity of a *Runx3*-reporter in CD8 SP thymocytes^43^. We also performed Bcl11b ChIP-seq in CD8-enriched thymocytes (mainly DP and CD8 SP thymocytes) and found Bcl11b binding at the −39 kb, −21 kb, and −18 kb *cis*-regulatory conserved sites, as well as at the P1 promoter (Fig. 7f), critical for expression of Runx3 in CD8^+^ T-cells^44^. Thus, in thymocytes, Bcl11b binds to the *cis*-regulatory regions located at −39 kb, −21 kb, and −18 kb and to the P1 promoter (Fig. 7f), while in naive and Th2 CD4^+^ T-cells Bcl11b binds only to the −39 kb and −18 kb conserved sites (Fig. 7f).

Finally, we evaluated Runx3 in CD4^+^ T-cells of mixed bone-marrow chimeric mice infected with *H. polygyrus bakeri*. We found that Runx3 was upregulated only in the CD4^+^ T-cells which had Bcl11b removed and not in WT cells (Fig. 7g, h), demonstrating that the upregulation of Runx3 in the absence of Bcl11b is intrinsic.

Thus these results demonstrate that Bcl11b directly restricts expression of *Runx3* in Th2 cells and in naive CD4^+^ T-cells by binding to and deactivating upstream *Runx3* enhancers.

## Discussion

In this study, we demonstrate that Bcl11b plays a critical role in the differentiation of Th2 cells in vivo. Bcl11b has been previously shown to repress GATA3 and IL-4 in Th17 cells during EAE^30^. In addition, Bcl11b^−/−^ CD4^+^ T-cells were shown to differentiate normally into Th2 cells in vitro, with their polarization being even slightly enhanced, compared to WT CD4^+^ T-cells^30^. However, in ILC2s, which are functionally similar to Th2 cells, Bcl11b was needed to sustain key type 2 transcription factors and cytokines and to block the ILC3 program^33^. We report here that Bcl11b is a critical transcription factor for licensing Th2 differentiation in vivo. Specifically, in response to allergen-induced asthma and intestinal helminth infection, CD4^+^ T-cells of *Bcl11b*^*fl/fl*^ dLck-iCre mice failed to efficiently differentiate into Th2 cells, despite the fact that they expanded normally. Consequently, *Bcl11b*^*fl/fl*^ dLck-iCre mice had reduced pulmonary pathology associated with the induction of allergic asthma, while having increased fecal egg burden following helminth infection. Transcriptome, chromatin accessibility and ChIP-seq analyses revealed the mechanisms by which Bcl11b regulated Th2 cell differentiation in vivo. In addition to the Th2 cytokines, GATA3 expression was reduced both at protein and mRNA levels. GATA3 has been shown to be indispensable for the initiation and propagation of Th2 differentiation^45^. The reduction in GATA3 was similar to the defects we previously found in the absence Bcl11b in ILC2s^33^, where, in addition, *Gfi1* and *Rora* were also downregulated. The reduction in GATA3 in ILC2s was shown to be dependent on diminished *Gfi1* expression. However, we did not find any changes in *Gfi1* expression in Bcl11b-deficient Th2 cells, suggesting that the control of Gfi1 by Bcl11b is cell-type dependent. We did find binding of Bcl11b to several conserved intronic regions of *Gata3* and non-coding sequences downstream of *Gata3* locus. Whether this binding has functional consequences, or if Bcl11b binds to these sites in ILC2s, remains to be established. The reduced IL-4 level in the absence of Bcl11b may also contribute to diminished *Gata3* expression, and it remains to be established what transcription factors mediate this regulation.

GATA3 itself is known to act at the Th2 LCR and at the *Il5 and Il13* gene promoters to enhance their transcription^46^. The Th2 LCR RHS V enhances *Il4* transcription through interactions with the *Il4* promoter, with GATA3 playing a central role in this process^10^. Bcl11b-ChIP-seq analysis showed no binding of Bcl11b at Th2-LCR, or Th2-cytokine promoters in Th2 cells; however, ATAC-seq showed reduced chromatin accessibility at the Th2 LCR RHS V and at the *Il5* and *Il13* promoters in the absence of Bcl11b. Thus the reduction in accessibility can be related, at least in part, to the decreased GATA3 levels in the absence of Bcl11b. In addition to the Th2 LCR and *Il4* promoter, *Il4* expression is also regulated through the *Il4* HS IV silencer, which functions to repress *Il4* expression in Th1 cells in vivo^11,12^. A second defect of Bcl11b^−^ T-helper cells during Th2 response was the high chromatin accessibility at the *Il4* HS IV silencer, much more than in WT Th2 cells, similar to Th1 cells. This increase was also seen in the naive state. We found that Bcl11b directly binds to *Il4* HS IV silencer in naive and CD4^+^ T-cells of mice infected with helminths, demonstrating that Bcl11b plays a critical role in directly limiting *Il4* HS IV silencer accessibility in Th2 and naive CD4^+^ T-cells. Previous studies have demonstrated that Runx3, normally expressed in Th1 cells and CD8^+^ T-cells, binds to the *Il4* HS IV silencer in Th1 cells and represses *Il4* expression^12^. In relation to this, Bcl11b^−^ T-helper cells from naive mice as well as from mice infected with helminths, had a third defect: showing a major elevation in Runx3 levels compared to Bcl11b-sufficient cells. In addition, Runx3 showed increased association with the *Il4* HS IV silencer in both Bcl11b^−^ CD4^+^ T-cells from naive and helminth-infected mice. Interestingly, the second motif that we identified as being bound by Bcl11b, “ACCACA”, appears to be a Runx binding motif^39^. Thus, absence of Bcl11b results in availability and increased accessibility of *Il4* HS IV silencer and elevated Runx3, which now can bind at the silencer. This, together with reduced levels of GATA3 cause diminished *Il4* expression and reduced ability to efficiently differentiate into Th2 lineage in vivo.

Runx3 has also been shown to be important in ILC1 and ILC3 development, but not in ILC2 development^44^. In ILC3s, Runx3 positively regulates *Rorc* expression, which in turn increases the expression of *Ahr*^44^. It remains to be established whether in Bcl11b-deficient ILC2s, Runx3 is derepressed in a manner similar to Bcl11b^−^ T-helper cells, which then may also contribute to the shift from ILC2 to ILC3 program^33^, a possibility which also exists in iNKT cells, where the absence of Bcl11b caused derepression of iNKT17 genes and reduction of the other iNKT cell subset genes^26^.

The disparities between the actions of Bcl11b on Th2 differentiation in vivo and in vitro have been of particular interest to us. We speculate that the constant supply of exogenous IL-4 during in vitro polarization is key to the observed changes, as previous reports have shown that certain defects in Th2 differentiation, such as those observed related to Notch signaling, can be compensated for in this way^4,47^. It is possible that by providing constant, high amounts of exogenous IL-4 during the in vitro polarization, a Bcl11b-deficient cell is able to gradually overcome its defect in IL-4 production and begins upregulating GATA3 to a level sufficient to compensate the defect, and the excess in Runx3 is eventually  overcame, allowing for the stabilization of the Th2 lineage in vitro.

We found that Bcl11b binds to several conserved *cis*-regulatory regions upstream of *Runx3*, namely at −39 kb, −21 kb, and −18 kb, as well as to the P1 promoter in CD8-enriched thymocytes, while in naive and Th2 CD4^+^ T-cells, it only bound to the −39 kb and −18 kb sites. Our finding that removal of Bcl11b results in enrichment of H3K27ac at the −39 kb and −18kb sites suggests that Bcl11b inactivates these enhancers in Th2 cells and likely in naive CD4^+^ T-cells. Very recently, in addition to our findings, Bcl11b was found to bind to the *Runx3* *cis*-regulatory regions −39 kb and −21 kb sites in total thymocytes^43^. When these regions were deleted, a reduction in *Runx3*-reporter expression was observed in CD8 SP thymocytes, indicating that these regions act as enhancers for *Runx3* in CD8 SP thymocytes^43^, and supporting the idea that in naive and Th2 cells, Bcl11b inactivates Runx3 enhancers, thus blocking its expression. It remains to be established whether Bcl11b binds to these *Runx3* *cis*-regulatory regions in DP, CD4 SP thymocytes, and/or CD8 SP thymocytes, given that the ChIP-seq analysis was conducted in total thymocytes^43^, and in CD8-enriched thymocytes (this study). Interestingly, Thpok was also found to bind to the −39 kb and −21 kb sites in total thymocytes^43^. We did not find any changes in Thpok mRNA levels in the absence of Bcl11b in Th2 cells. Thus *Runx3* repression in mature naive and Th2 CD4^+^ T-cells requires Bcl11b, and Thpok alone is likely not sufficient to exert this function. However, Thpok is essential in blocking Runx-dependent differentiation toward CD8 lineage^48^. We did not notice any increase in CD8^+^ T-cells versus CD4^+^ T-cells as a consequence of Runx3 upregulation, likely due to the fact that removal of Bcl11b occurred after commitment to CD4 SP and CD8 SP thymocytes. In addition, CD8 expression was not detected in CD4^+^ T-cells. It remains to be established how Bcl11b regulates Runx3 in Th1 cells and CD8^+^ T-cells, where Runx3 is expressed^49^.

Similar to ILC2s, in Th2 cells, Bcl11b is also required to repress type 3 program including *Rorc* and *Il17*, however different from ILC2s, the proteins could not be detected in Bcl11b^−^ Th2 cells, thus unlikely to participate in the phenotype. *Tbx21* and *Ifng* mRNAs, but not proteins, were also upregulated. Except for *Il17, Ifng*, and *Runx3*, Bcl11b did not bind any other Th17 or Th1 loci or adjacent non-coding regions.

Bcl11b also repressed expression of several myeloid genes, including those encoding Cd11b, Cd11c, and Fc receptors, as well as of some killer cell lectin-like receptors and also bound to *cis*-regulatory regions within or upstream of these genes, suggesting that Bcl11b may directly repress their expression in differentiating Th2 cells.

In conclusion, we established here that the absence of Bcl11b in naive and during Th2 response in vivo results in derepression of Runx3, as well as provision of access for Runx3 to the *Il4* HS IV silencer. These events, together with reduced GATA3, caused reduced chromatin accessibility at Th2 LCR, and Th2 gene promoters, further resulting in reduced Th2 differentiation. These results together suggest that Bcl11b primes naive CD4^+^ T-cells in such a way to be permissive for Th2 differentiation, and absence of Bcl11b causes a failure in efficient licensing of Th2 lineage.

## Methods

### Mice

*Bcl11b*^*fl/fl*^ dLck-iCre mice on a C57BL/6 were previously described^28^. All mice were raised and housed in pathogen-free conditions in the University of Florida Animal Facilities except mice infected with *H. polygyrus*, which were transferred to a BSL2 facility prior to infection. All experiments were conducted on 8–12-week-old male and female mice. All mouse work was approved by the University of Florida Institutional Animal Care and Use Committee. No statistical methods were used to predetermine sample size.

### Induction of allergic asthma by HDM extract

8–12-week-old mice were sensitized to house-dust-mite antigen by seven intraperitoneal injections of 100 μL PBS containing 50 µg HDM extract (*D. pteronyssinus*, Greer Labs) on days 1, 3, 5, 7, 9, 11, and 13. Then, on days 25, 26, and 27, mice were challenged intranasally (i.n.) with 20 μL PBS containing 50 µg HDM extract. Mice were anesthetized with isoflurane prior to each i.n. challenge. Mice were sacrificed on day 29.

### Lung digestion for isolation of leukocytes

Lungs were initially perfused through the right ventricle with 10 mL cold PBS. The lungs were then excised and placed in a C tube (Miltenyi Biotech) containing 5 mL cold PBS and subsequently minced on a gentleMACS Dissociator (Miltenyi Biotech). The lungs were then digested for 45 min at 37 °C with the addition of 0.2 Wünsch units/mL of Liberase TM (Roche) and 0.15 mg/mL DNase I (Roche). Digested tissue was filtered through a 70-μm strainer and washed with complete RPMI. Epithelial cells were removed by density separation using a 30%/70% discontinuous Percoll gradient.

### Helminth infection

8–12-week-old mice were infected with *H. polygyrus* via oral gavage with 200 μL water containing 200 L3 larvae. Fecal pellets were collected immediately before euthanasia on day 12 post infection, with the eggs/g feces serving as a readout of overall worm fitness.

### Isolation of leukocytes from the SILP

Small intestinal lamina propria preparation methodology was adapted from previous study^50^. Small intestines were flushed with cold PBS and PPs were removed and set aside. Tissue was cleaned and washed once with 5 mM DTT and thrice with 5 mM EDTA. SILP was then digested at 37 °C for 30 min with 0.2 Wünsch units/mL of Liberase TM (Roche) and 0.1 mg/mL DNase I (Roche). Digested tissue was filtered through a 70-μm strainer and washed with complete RPMI. Remaining epithelial cells were removed by density separation using a 30%/70% discontinuous Percoll gradient.

### Isolation of CD4^+^ T-cells from total leukocytes

For leukocytes prepared from lung tissue, CD4^+^ T-cells were purified by FACS, using a BioRad S3e Cell Sorter. For cells from the mesenteric and mediastinal lymph nodes, CD4^+^ T-cells were purified using Dynabeads Untouched Mouse CD4 Cell kit (Invitrogen).

### Enrichment of IL-4^+^CD4^+^ T-cells from total leukocytes

Labeling and enrichment of IL-4^+^ cells from pre-negatively selected CD4^+^ T-cells was performed using the Mouse IL-4 Secretion Assay kit from Miltenyi Biotech (#130-00-515). Enrichment was verified to be 40%, by flow cytometry.

### RNA-Seq

RNA was extracted from CD4^+^ T-cells purified from the mLNs of *H. polygyrus* infected mice or the mediLNs of asthmatic mice using RNeasy Plus Micro Kit (QIAGEN). cDNA libraries were prepared off-site and sequencing was performed on a NextSeq 500. Sequencing reads were processed and analyzed using a custom pipeline. Fastq files were initially trimmed using seqtk^51^. Quality of the fastq files were assessed via FastQC^52^. Trimmed reads were aligned to the mouse (mm10) genome using Hisat2^53^. Htseq^54^ was utilized to obtain transcript counts for each sample, which were then run through DeSeq2^55^ for differential analysis.

### ATAC-Seq

DNA from 50,000 CD4^+^ T-cells was extracted and processed for ATAC-Seq as described by others^40^. Library quality and adapter dimer contamination was assessed on a Bioanalyzer (Agilent, #5067-4626). Libraries were run as PE75 on a NextSeq 500. Data analysis was performed using a custom pipeline based on a modified version of a previously published method^56^. Fastq files were initially trimmed using Trimmomatic^57^, then read quality was assessed using FastQC as before^52^. Reads were aligned to the mouse (mm10) genome using Bowtie2^58^ and the resulting SAM file was pruned (*Q* > 30), converted into BAM format, and sorted using SAMtools^59^. Duplicate reads were identified and removed using Picard tools^60^. Peaks were called and bedgraph files were prepared using MACS2 peak calling software^61^. Bedgraph files were then visualized using Integrative Genomics Viewer (IGV)^62^.

### Bcl11b ChIP-Seq

ChIP-Seq against Bcl11b was performed on CD4^+^ T-cells using the SimpleChIP Enzymatic Chromatin IP Kit from Cell Signaling Technologies (#9003), following their recommended protocol up to the library preparation with the following alterations: (1) 10^7^ cells were used in place of 4 × 10^7^; (2) formaldehyde fixation was performed in 1 mL; (3) Buffer A and Buffer B steps were all performed in 1 mL; (4) 2 μl of 1:10 diluted Micrococcal nuclease was determined to give the optimum digestion results by titration; (5) cocktail of two anti-Bcl11b antibodies were used (8 μg ab18465 and 3 μg of CST #12120 per ChIP); (6) final DNA purification was performed by phenol:chloroform:isoamyl alcohol extraction with MaXtract high density columns (Qiagen #129046). Libraries were prepared using NEBNext DNA Library Kit (NEB, #E7645), with quality being determined as above. Libraries were run as SE75 on a NextSeq 500. Fastq analysis performed as with ATAC-Seq, but with single-end rather than paired-end settings.

### H3K27ac ChIP-Seq

ChIP-Seq against H3K27ac was performed on 100,000 CD4^+^ T-cells isolated from the lungs of HDM asthma-induced *Bcl11b*^*fl/fl*^ dLck-iCre and *Bcl11b*^*fl/fl*^ mice using the Low Cell ChIP-Seq kit from Active Motif (#104895), following their recommended protocol scaled to 100,000 cells. The antibody used was acetyl-histone H3 (Lys27) (D5E4) XP® Rabbit mAb from Cell Signaling Technologies (#8173) at the recommended 1:100 dilution. Libraries were run as SE75 on a NextSeq 500. Fastq analysis performed as with Bcl11b ChIP-Seq.

### Mixed bone-marrow chimeras

To generate mixed bone-marrow chimeras, CD45.1 mice were irradiated twice with 500 rads, 4 h apart, on the same day prior to reconstitution with cells from CD45.1/CD45.2 *Bcl11b*^*fl/fl*^ mice and CD45.2 *Bcl11b*^*fl/fl*^ dLck-iCre mice at a 1:1 ratio. Recipient mice were then left for a minimum of 8 weeks (the first two of which were on antibiotics), at which point they were used for experimentation.

### Cytokine stimulation for intracellular flow cytometry

For detection of cytokines by intracellular flow cytometry, cells were cultured at 37 °C and 5% CO_2_ for 4 h in IMDM media containing PMA (20 ng/ml) and Ionomycin (1 µg/ml). Brefeldin A (10 µg/ml) was added after 1 h. After incubation, cells were washed and stained with Fixable viability dye (Affymetrix) and surface markers. Cells were then fixed and permeabilized with Foxp3 Fix/Perm kit (Affymetrix, #00-5521), then subsequently stained for cytokines.

### Flow cytometry

Flow cytometry was performed on an BD LSR II, with data acquired using BD FACS DIVA software. All data were analyzed using FlowJo (TreeStar).

### Antibodies

The antibodies used either for staining for flow cytometry or for ChIP-seq and ChIP-PCR are shown in Table 1.

**Table 1:** Antibodies used in this study

Target	Clone	Source	Identifier	Concentration
Anti-mouse CD4	GK1.5	Affymetrix	48-0041	0.25 µg/100 μl
Anti-mouse CD4	GK1.5	BioLegend	100453	0.25 µg/100 μl
Anti-mouse CD8a	53-6.7	Affymetrix	25-0081	0.5 µg/100 μl
Anti-mouse CD8a	53-6.7	Affymetrix	47-0081	0.5 µg/100 μl
Anti-mouse CCR9	eBioCW-1.2	Affymetrix	11-1991	0.25 µg/100 μl
Anti-mouse CD44	IM7	Affymetrix	48-0441	0.25 µg/100 μl
Anti-mouse CD62L	MEL-14	Affymetrix	11-0621	0.5 µg/100 μl
Anti-mouse α4β7	DATK32	Affymetrix	17-5887	0.25 µg/100 μl
Anti-mouse CD11b	M1/70	Affymetrix	17-0112	0.125 µg/100 μl
Anti-mouse CD11c	N418	Affymetrix	12-0114	0.5 µg/100 μl
Anti-mouse GR-1	1A8-Ly6g	Affymetrix	11-9668	0.5 µg/100 μl
Anti-mouse Ly6C	HK1.4	Affymetrix	48-5932	0.25 µg/100 μl
Anti-mouse SiglecF	E50-2440	BD Pharmingen	562680	0.2 µg/100 μl
Anti-mouse IL-4	11B11	BioLegend	504125	0.4 µg/100 μl
Anti-mouse IL-5	TRFK5	Affymetrix	12-7052	0.25 µg/100 μl
Anti-mouse IL-13	eBio13A	Affymetrix	53-7133	0.25 µg/100 μl
Anti-mouse Gata3	L50-823	BD Pharmingen	560068	20 µl/100 μl
Anti-mouse Gata3	16E10A23	BioLegend	653808	5 µl/100 μl
Anti-mouse Rorγ(t)	AFKJS-9	Affymetrix	12-6988	0.5 µg/100 μl
Anti-mouse T-bet	4B10	BioLegend	644816	0.4 µg/100 μl
Anti-mouse Ki67	SolA15	Affymetrix	11-5698	0.25 µg/100 μl
Anti-mouse Runx3	R3-5G4	BD Pharmingen	564814	5 µl/100 μl
Fixable viability dye	N/A	Affymetrix	65-0865	0.1 µl/100 μl
Anti-mouse Runx3	Polyclonal	See ref.^63^	N/A	5 μl/1000 μl (ChIP)
Anti-mouse Ctip2	25B6	abcam	ab18465	8 μg/1000 μl (ChIP)
Anti-mouse Bcl11b	D6F1	Cell Signaling Technologies	12120	3 μg/1000 μl (ChIP)

### Statistical analyses

For non-sequencing experiments, differences between groups were calculated by a two-tailed Student’s *t*-test assuming unequal variance. For RNA-Seq experiments, significance was calculated by DeSeq2 with a false discover rate (FDR) cutoff of 0.05. For ATAC-Seq experiments, significant differences in enrichment between peaks was determined using an optimized MANorm script^64,65^ with the cutoff set at a minimum fold-change of 2 and significance of *p* *<* 0.05.

### Data availability

RNA-seq, ChIP-seq, and ATAC-seq data shown in this study have been deposited in the Gene Expression Omnibus (GEO) database with accession GSE99599 (https://www.ncbi.nlm.nih.gov/geo/query/acc.cgi?acc = GSE99599).

All other relevant data and reagents are available from the authors.

## Electronic supplementary material

Supplementary Information
