# Publisher Correction: Bcl11b is essential for licensing Th2 differentiation during helminth infection and allergic asthma

**DOI:** 10.1038/s41467-018-05360-9

**Published:** 2018-07-19

**Authors:** Kyle J. Lorentsen, Jonathan J. Cho, Xiaoping Luo, Ashley N. Zuniga, Joseph F. Urban, Liang Zhou, Raad Gharaibeh, Christian Jobin, Michael P. Kladde, Dorina Avram

**Affiliations:** 1 Department of Medicine, Division of Pulmonary Medicine, College of Medicine, University of Florida, 1600 SW Archer Rd, 32610 Gainesville, FL USA; 2 Department of Anatomy and Cell Biology, University of Florida College of Medicine, 32610 Gainesville, FL USA; 3 Beltsville Human Nutrition Research Center, Agricultural Research Service, Diet, Genomic and Immunology Laboratory, US Department of Agriculture, 20705 Beltsville, MD USA; 4 Department of Infectious Diseases and Immunology, College of Veterinary Medicine, University of Florida, 2015 SW 16th Ave, 32608 Gainesville, FL USA; 5 UF Health Cancer Center, University of Florida, 32610 Gainesville, FL USA; 6 Department of Medicine, Division of Gastroenterology, College of Medicine, University of Florida, 2033 Mowry Rd, CGRC 461, 32610 Gainesville, FL USA; 7 Department of Biochemistry and Molecular Biology, College of Medicine, University of Florida, 2033 Mowry Rd., CGRC 359, 32610 Gainesville, FL USA

Correction to: *Nature Communication;* 10.1038/s41467-018-04111-0, published online 26 April 2018

In the originally published version of this Article, the affiliation details for Dorina Avram incorrectly included “Department of Infectious Diseases and Immunology, College of Veterinary Medicine, University of Florida, 2015 SW 16th Ave, Gainesville, FL, 32608, USA”, instead of “UF Health Cancer Center, University of Florida, Gainesville, FL 32610, USA”. This has now been corrected in both the PDF and HTML versions of the Article.

