# Supplementary material for: Bcl11b is essential for licensing Th2 differentiation during helminth infection and allergic asthma
Source: Nat Commun. 2018 Apr 26;9:1679. doi: 10.1038/s41467-018-04111-0 (PMC5920086; doi:10.1038/s41467-018-04111-0)
Supplement: Supplementary file 1 — Supplementary Information [file 41467_2018_4111_MOESM1_ESM.pdf]

Bcl11b is essential for licensing Th2 differentiation during helminth infection and allergic asthma

Lorentsen et al.

SUPPLEMENTARY FIGURES AND FIGURE LEGENDS

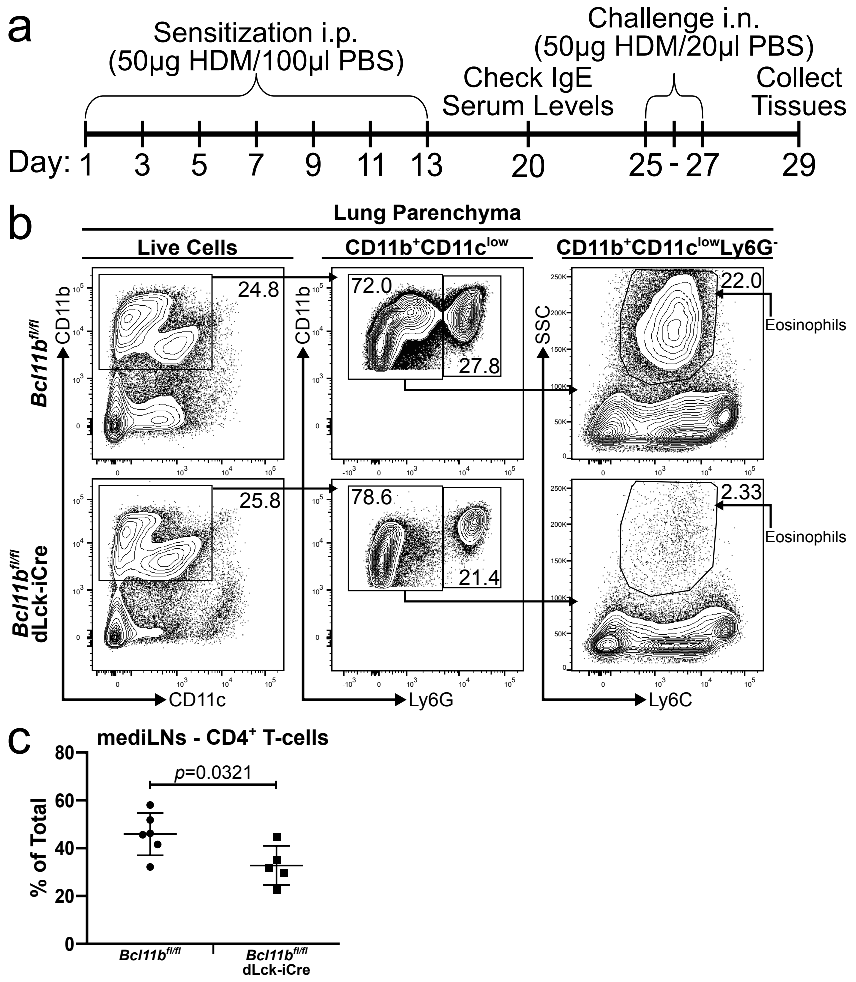

Supplementary Figure 1 (related to Fig 1). Allergic asthma induced with house dust mite (HDM) extract in *Bcl11b*$^{fl/fl}$ dLck-iCre and control mice (a) Description of the method used to induce HDM-dependent allergic asthma in mice. (b) Flow cytometry analysis of eosinophils. CD11b^{+}CD11c^{low} (left column), CD11b^{+}CD11c^{low}Ly6G^{-} (central column) and CD11b^{+}CD11c^{low}Ly6G^{-}Ly6C^{mid}SSC^{hi} (right column) populations in the lung parenchyma of HDM asthma-induced mice. (c) Frequency of CD4^{+} T-cells in the mediastinal lymph nodes of the indicated groups of HDM asthma-induced *Bcl11b*$^{fl/fl}$ dLck-iCre mice (n=6) and *Bcl11b*$^{fl/fl}$ control mice (n=5). Data are representative of three independent experiments. All statistical values in this figure were calculated using Student's *t* test. All error bars shown in this figure are of standard deviation.

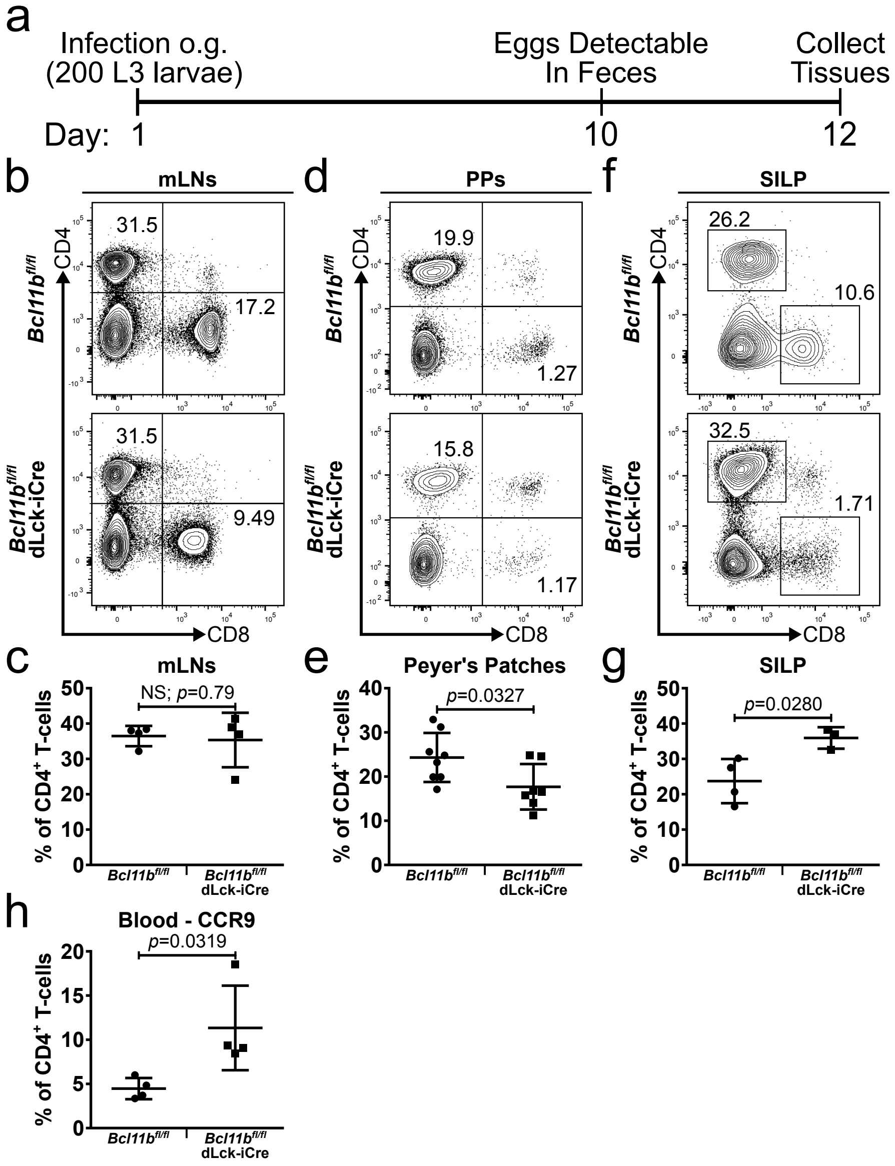

Supplementary Figure 2 (related to Fig 2). ***Bcl11b**$^{fl/fl}$* **dLck-iCre mice have altered frequencies of CD4^{+} T-cells during helminth infection and increased CCR9 on peripheral blood CD4^{+} T-cells** (a) Description of the method used to infect mice with *H. polygyrus bakeri*. (b, d, f) Flow cytometry analysis of the CD4^{+} and CD8^{+} T-cell populations in the mesenteric lymph nodes (b), Peyer's patches (d), and small intestinal lamina propria (f) of helminth infected mice from the indicated groups at 12 days post-infection. (c, e, g) Frequencies of CD4^{+} T-cells in the mesenteric lymph nodes (c) (n=4), Peyer's patches (e) (n=8), and SILP (g) (n=3-4) of helminth infected mice from the indicated groups. Data are representative of 3 independent experiments. Significance was determined by Student's *t* test. (h) Frequencies of CCR9^{+} CD4^{+} T-cells in the blood. Data are

representative of two independent experiments (n=4). All statistical values in this figure were calculated using Student's *t* test. All error bars shown in this figure are of standard deviation.

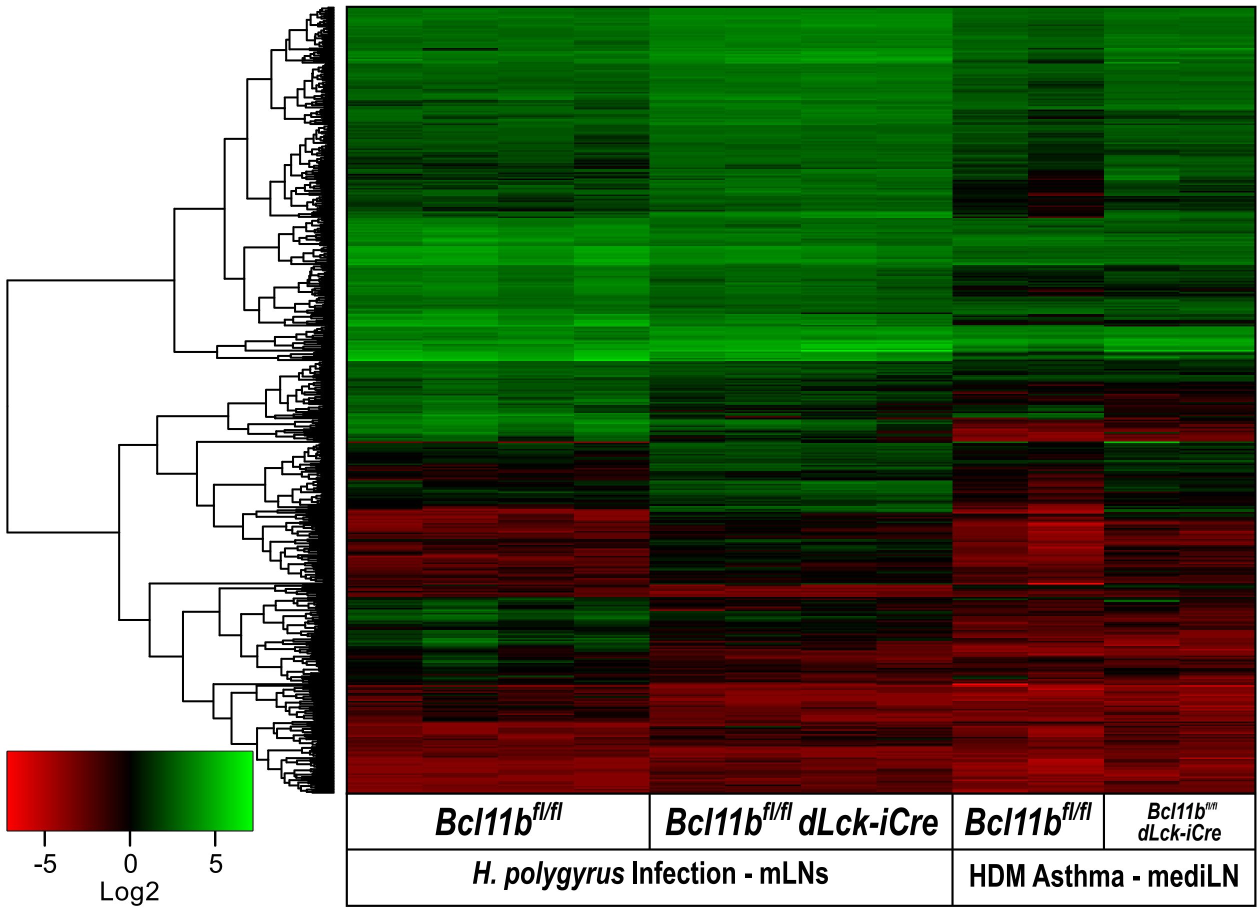

Supplementary Figure 3 (related to Fig 3). Heatmap of RNA-seq data from CD4$^+$ T-cells isolated from the mesenteric lymph nodes (mLNs) of *H. polygyrus bakeri* infected *Bcl11b$^{fl/fl}$* dLck-iCre and control mice (left) and from mediastinal lymph nodes (mediLNs) of HDM-induced asthmatic mice (right) Heatmap was generated in R.

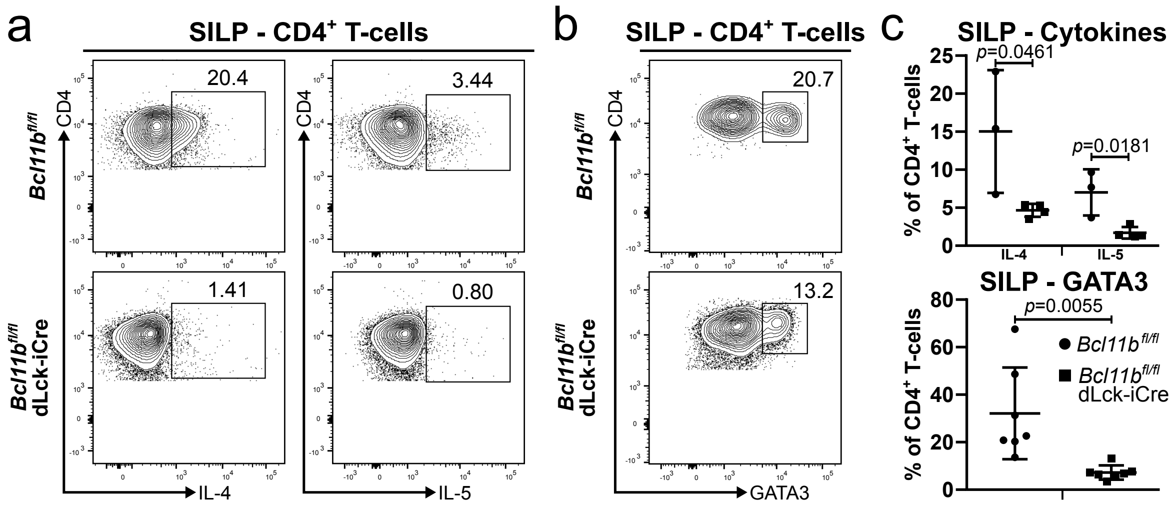

Supplementary Figure 4 (related to Fig 4). GATA3 and Th2 cytokines produced by Bcl11b-deficient and wild type CD4$^+$ T-cells from the small intestine lamina propria (SILP) of helminth-infected *Bcl11b$^{fl/fl}$* dLck-iCre mice and *Bcl11b$^{fl/fl}$* control mice (a) Flow cytometry analysis of IL-4 (left column) and IL-5 (right column) levels in the CD4$^+$ T-cell population in the (SILP) of the indicated groups of *H. polygyrus bakeri* infected mice. (b) Flow cytometry analysis of GATA3 within CD4$^+$ T-cell population in the SILP of the indicated groups of *H. polygyrus bakeri* infected mice. (c) Frequencies of IL-4$^+$, IL-5$^+$ (top panel, n=3-4) and GATA3$^+$ (bottom panel, n=7) CD4$^+$ T-cells from the SILP of *H. polygyrus bakeri* infected mice from the indicated groups. Data are derived from two (IL-4 and IL-5 data) or four (GATA3 data) independent experiments. All statistical values in this figure were calculated using Student's *t* test. All error bars shown in this figure are of standard deviation.

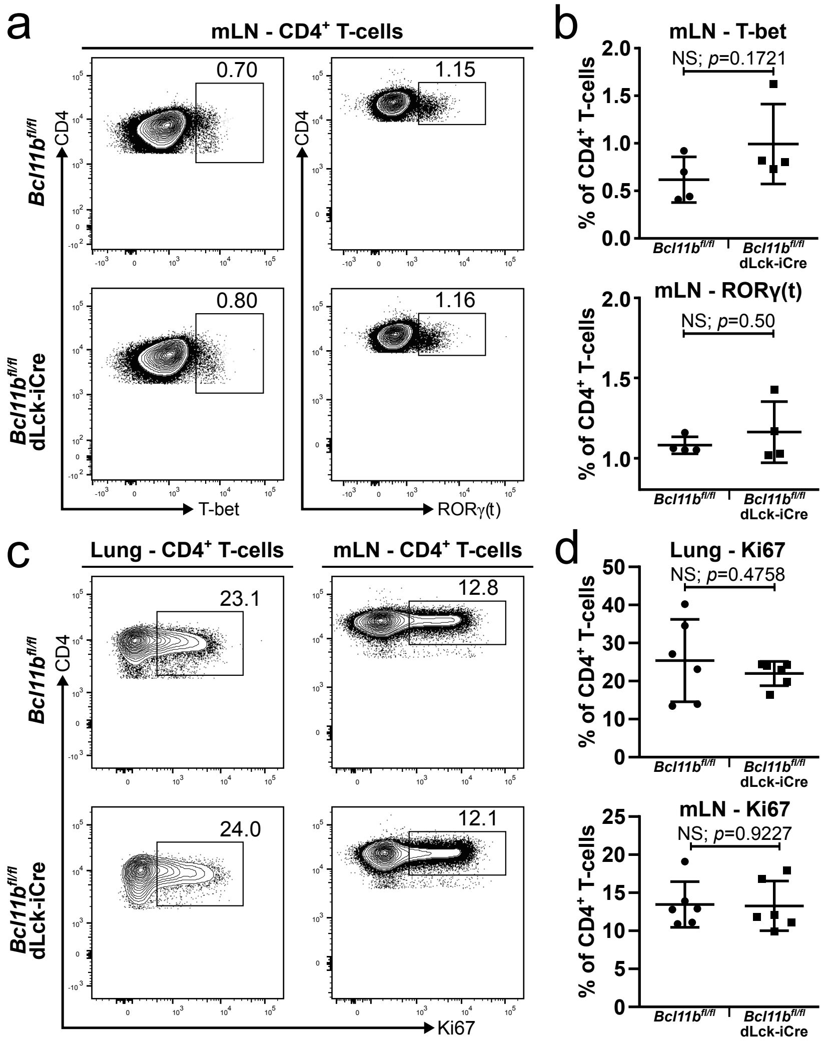

Supplementary Figure 5 (related to Fig 5). Bcl11b-deficient CD4$^+$ T-cells have normal T-bet and RORγ(t) levels and proliferate normally (a) Flow cytometry analysis of T-bet (left column) and RORγ(t) (right column) in CD4$^+$ T-cells from the mesenteric lymph nodes of helminth infected *Bcl11b*$^{fl/fl}$ dLck-iCre mice and *Bcl11b*$^{fl/fl}$ control mice. (b) Frequencies of T-bet$^+$ (top) and RORγ(t) (bottom) cells within the CD4$^+$ T-cell population from the mesenteric lymph nodes of helminth infected mice from the indicated groups. Data are representative of two independent experiments (n=4). (c) Flow cytometry analysis of Ki67 within the CD4$^+$ T-cell population of the lung parenchyma of HDM asthma-induced mice (left column) and from the mesenteric lymph nodes of H. polygyrus bakeri infected mice (right column) from the indicated groups. (d) Frequencies of Ki67+ cells within the CD4$^+$ T-cell population in the lung parenchyma of HDM asthma-induced mice (top) (n=6) and the mesenteric lymph nodes of helminth infected mice (bottom) (n=6). Data are representative of 3 independent experiments. Significance was determined by Student's t test. All error bars shown are of standard deviation.

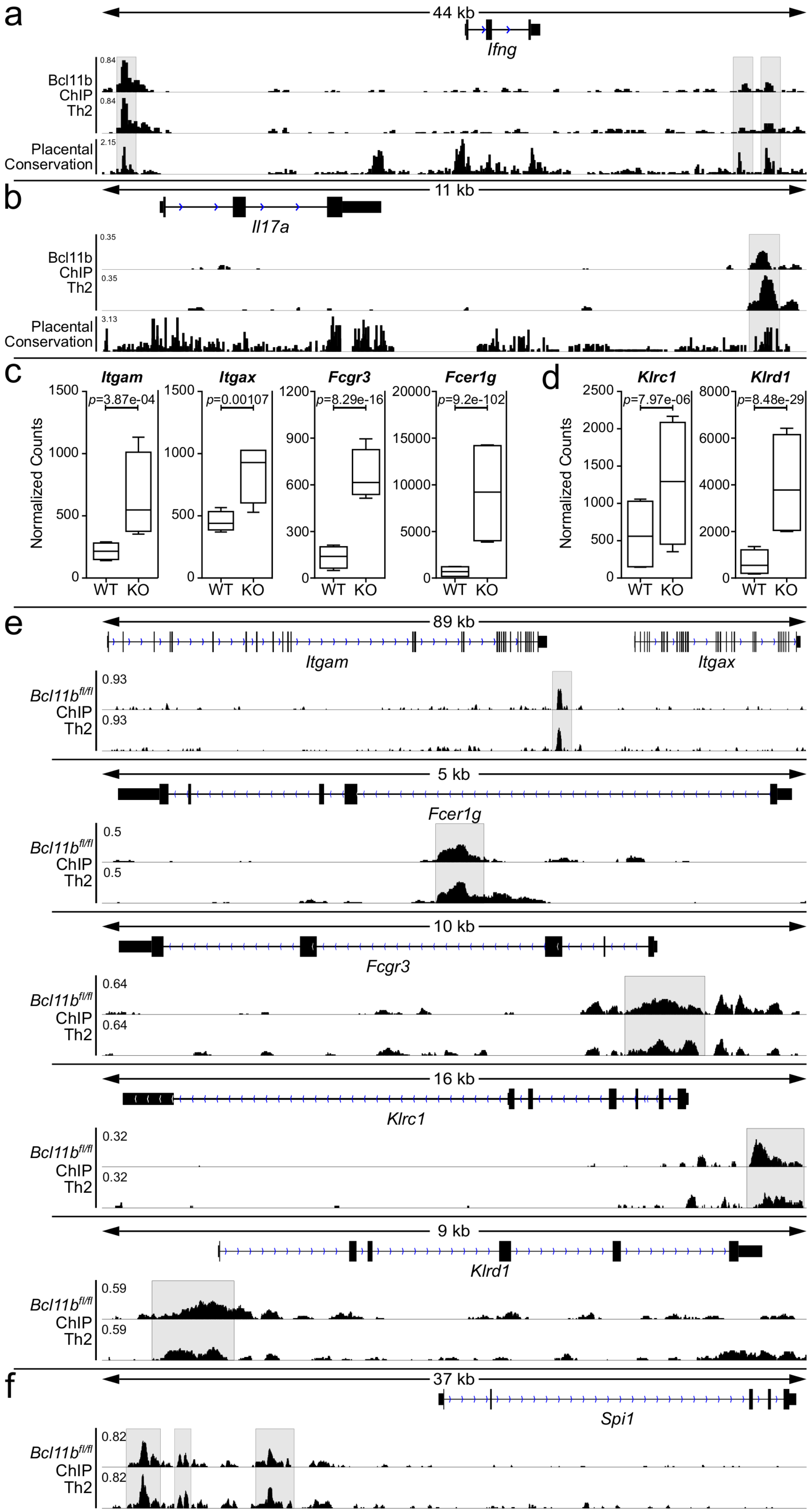

a
44 kb
Ifng
Bcl11b ChIP Th2
Placental Conservation
b
11 kb
Il17a
Bcl11b ChIP Th2
Placental Conservation
c
Itgam
Itgax
Fcgr3
Fcer1g
Normalized Counts
p=3.87e-04
p=0.00107
p=8.29e-16
p=9.2e-102
WT KO
d
Klrc1
Klrd1
p=7.97e-06
p=8.48e-29
e
89 kb
Itgam
Itgax
Bcl11bfl/fl ChIP Th2
5 kb
Fcer1g
10 kb
Fcgr3
16 kb
Klrc1
9 kb
Klrd1
f
37 kb
Spi1

Supplementary Figure 6 (related to Fig 6). Bcl11b binds to and regulates expression of certain Th1 Th17 myeloid and NK genes in CD4⁺ T-cells during helminth infection (a-b) Integrative Genomics Viewer visualizations of Bcl11b ChIP-Seq tracks at *Ifng* (a) and *Il17a* (b). (c-d) Box and whisker plots of normalized counts for myeloid- (c) and NK- (d) associated genes. Normalized counts and statistical analyses were taken from DESeq2 analysis software. Data is derived from two independent experiments. *p* values have been adjusted for the FDR (false discovery rate) by the Benjamini-Hochberg adjustment. All error bars shown are of standard deviation. (e) Integrative Genomics Viewer visualizations of Bcl11b ChIP-Seq tracks at the *Itgam/Itgax* locus, *Fcer1g* , *Fcgr3* , *Klrc1, and Klrd1*. (f) Integrative Genomics Viewer visualizations of Bcl11b ChIP-Seq tracks at the *Spi1* locus. All ChIP-Seq track scale bars normalized to sequences per million reads (SPMR).

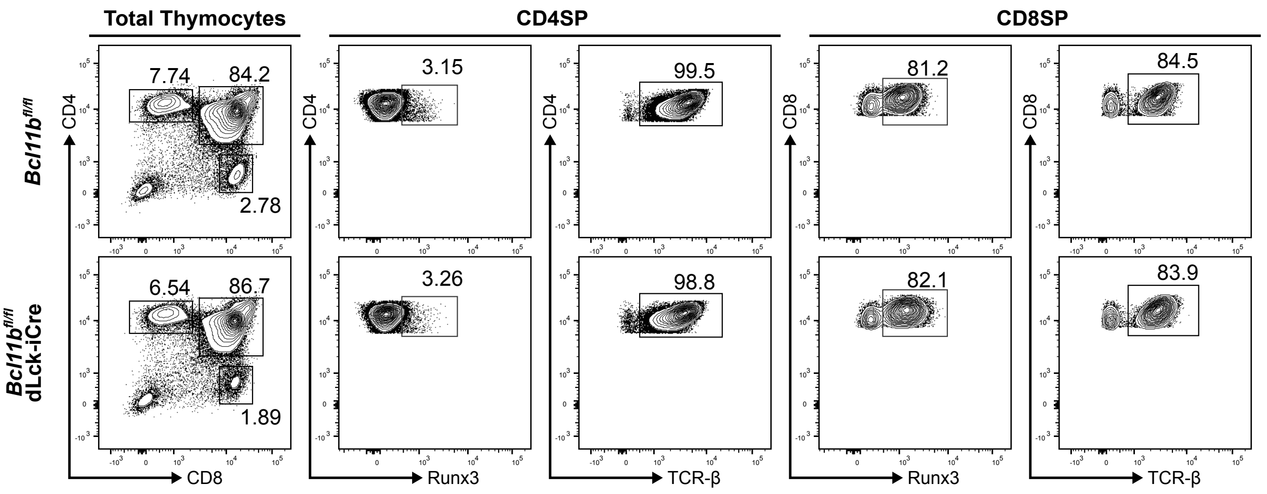

Figure S7 (related to Fig 7). Runx3 and TCRβ in CD4SP and CD8SP thymocytes of *Bcl11b*$^{fl/fl}$ dLck-iCre and *Bcl11b*$^{fl/fl}$ mice Flow cytometry analysis of Runx3 and TCR-β in the CD4SP and CD8SP thymocytes from naïve *Bcl11b*$^{fl/fl}$ (n=6) and *Bcl11b*$^{fl/fl}$ dLck-iCre (n=6) mice. Data are representative of two independent experiments.
